# Identification and Fine-Mapping of *qBr10*, a Major-Effect Locus for Shoot Branching in Sunflower (*Helianthus annuus*)

**DOI:** 10.3390/ijms27093715

**Published:** 2026-04-22

**Authors:** Mingzhu Zhao, Dianxiu Song, Xiaohong Liu, Bing Yi, Yuxuan Cao, Jingang Liu, Dexing Wang, Liangshan Feng

**Affiliations:** 1 Institute of Crop Research, Liaoning Academy of Agricultural Sciences, Shenyang 110161, China; zhaomingzhu23@163.com (M.Z.);; 2 Liaoning Academy of Agricultural Sciences, Shenyang 110161, China

**Keywords:** *Helianthus annuus*, shoot branching, BSA-seq, QTL fine-mapping, KASP

## Abstract

Shoot branching, as an important architectural trait, influences the number of flower heads and the pattern of flowering in sunflowers (*Helianthus annuus* L.). However, the main genetic factors leading to extensive branching throughout the plant were not clearly understood. In this study, we analyzed branching inheritance and identified a significant locus using an F_2_ population (*n* = 660) from a cross between the non-branched line 150A and the highly branched line PT326. The branching phenotypes varied from having no branches to complete plant branching, with segregation fitting a 3:1 ratio (χ^2^ = 2.916, *p* > 0.05), suggesting that a single major gene controls this trait, with the non-branched phenotype being dominant. Using bulked segregant analysis (BSA) and whole-genome resequencing, a strong and consistent signal was identified on chromosome 10 across three separate statistical analyses, pinpointing a primary candidate interval of approximately 3.40 Mb, named *qBr10*. Through the use of 10 developed Kompetitive Allele-Specific PCR (KASP) markers and recombinant screening, *qBr10* was restricted to a 388.5 kb (Chr10:13,422,378–13,780,875). Analysis of this interval identified 21 genes, among which *WRKY21* and *MTB3* were prioritized as candidate genes for further functional validation. Our findings identified *qBr10* as a strong candidate for cloning and offer closely associated markers to aid in marker-assisted improvement of branching and capitulum number in sunflower breeding.

## 1. Introduction

The productivity and value of sunflower (*Helianthus annuus*), a key oilseed and ornamental plant, are significantly influenced by its plant structure. The branching of shoots is crucial for plant architecture as it influences the quantity, placement, and efficiency of lateral branches. In sunflowers, branching is significant because each branch can end in a capitulum, so the number of heads, flowering synchrony, source-sink balance, and ultimately the harvest index and hybrid seed production efficiency are directly affected by branch initiation and outgrowth [1,2]. Through domestication and improvement, the modern cultivated sunflower has developed a strong apical dominance and a single large terminal head, in contrast to wild populations that often display extensive branching with multiple heads [3,4,5]. Even with the obvious phenotypic variation, the genetic basis for branching in sunflowers remains partially unresolved, and is usually complicated by inheritance that depends on the background, multiple types of branching (basal, apical, or whole-plant), and interactions between genotype and environment [6,7].

Research on both model and crop systems has shown that axillary bud activity is controlled by a combined network of hormonal and environmental factors. Auxin-mediated apical dominance, cytokinin-induced bud activation, and strigolactone-mediated inhibition of bud growth are fundamental components of branching regulation. Within buds, transcriptional hubs such as the TEOSINTE BRANCHED1/CYCLOIDEA/PROLIFERATING CELL FACTOR (TCP) factor BRANCHED1/TEOSINTE BRANCHED1 (BRC1/TB1) work to merge these signals [8,9,10,11]. However, there have been continuous challenges in adapting this conserved framework for sunflowers. Many studies on sunflowers have identified genomic regions associated with branching; however, the majority of loci demonstrate small to moderate effects and are mapped with limited accuracy, which hampers the discovery of causal genes and their implementation in breeding [7,12].

This study overcomes these limitations by using a major-effect segregation for whole-plant branching in an F_2_ population and integrating bulked segregant analysis sequencing (BSA-seq) with high-density marker-based fine-mapping. BSA-seq speeds up the identification of loci by comparing allele-frequency patterns between extreme groups and is a reliable method when the experimental setup manages bulk size, sequencing depth, and marker quality [13,14]. Here, three complementary statistics (ED, G′, and Index-slid) converged on a chromosomal locus *qBr10*, which is subsequently narrowed to a 388.5 kb interval *via* recombinant screening with Kompetitive Allele-Specific PCR (KASP) markers. By integrating interval annotation and stem-relevant expression evidence, we further prioritize *WRKY21* and *MTB3* as the most plausible causal candidates underlying whole-plant branching.

## 2. Results

### 2.1. Segregation of Branching Phenotypes in the F_2_ Population

**Figure 1:**
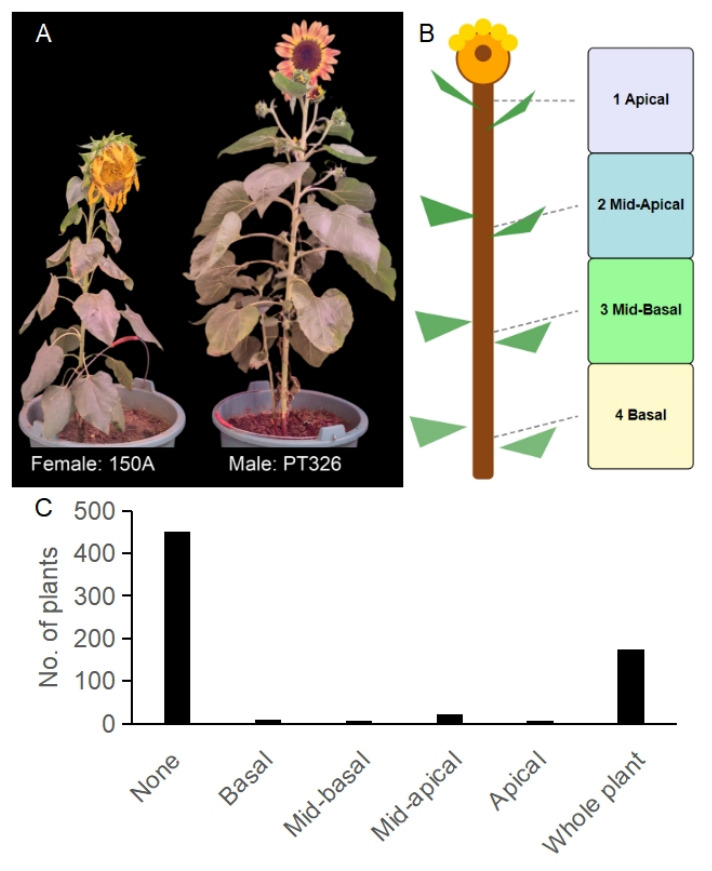
Phenotype of branching in sunflower. (**A**) Representative images of the parental lines: 150A (non-branched, left) and PT326 (highly branched, right). (**B**) Examples of branching pattern categories observed in the F_2_ progeny, ranging from no branches (None) to branching across the entire plant (Whole-plant branching), as well as intermediate patterns: Basal, Mid-basal, Mid-apical, and Apical. (**C**) Frequency distribution of F_2_ individuals across the different branching pattern categories (*n* = 660).

The parent line 150A exhibited a single-headed and non-branched phenotype, while PT326 had a multi-headed and highly branched phenotype (Figure 1A). F1 plants derived from the cross between these two parental lines were self-pollinated to generate the F2 population used for segregation analysis and mapping. In the F_2_ population from the cross of 150A and PT326, diverse branching phenotypes were observed, such as None, Basal, Mid-basal, Mid-apical, Apical, and Whole-plant branching (Figure 1B). There were 451 None plants, 10 Basal plants, one Mid-basal plant, 22 Mid-apical plants, one Apical plant, and 175 Whole-plant branching plants (Figure 1C). When the F_2_ progeny were classified into two major phenotypic classes (None vs. Whole-plant branching), the segregation pattern was consistent with a 3:1 ratio expected for a single major-effect locus. Based on the observed counts shown in Table 1, the chi-square goodness-of-fit test gave χ^2^ = 2.916, with 1 degree of freedom, corresponding to an exact *p*-value of 0.0877. Thus, the null hypothesis of 3:1 segregation was not rejected at the 0.05 significance level, indicating no significant segregation distortion from the expected Mendelian ratio. The observed proportion of Whole-plant branching individuals was 27.96%, with an approximate 95% confidence interval of 24.6–31.6%, which includes the expected 25.0% under a 3:1 model. These findings indicated that a single major-effect locus controls the primary branching trait in this cross, with the non-branched (None) phenotype being dominant over the branched (Whole-plant branching) phenotype.

### 2.2. BSA Identifies a Major-Effect Locus on Chromosome 10

To quickly identify the main locus, two extreme groups were formed by combining 30 None-type and 30 Whole-plant branching-type F_2_ individuals, along with both parents, for whole-genome resequencing. High-quality resequencing data were obtained for both parents and the two bulks (Table S2). Clean data ranged from 74.76 to 107.82 Gb, with Q30 values of 96.30–96.93%, average sequencing depths of 27.55×–37.76×, mapping rates of 99.69–99.81%, and genome coverage of 87.37–94.63%. These metrics indicated sufficient sequencing depth and broad, relatively uniform representation of the reference genome across samples, allowing for comprehensive genome-wide variant discovery and subsequent association analysis. Single Nucleotide Polymorphisms (SNPs) and Insertions and Deletions (InDels) were detected and compiled (Tables S3–S6), offering extensive marker coverage throughout the genome. A total of 87,943 SNPs and 10,478 InDels were identified in BSA, which were informative for the parents and fixed for different homozygous alleles between 150A and PT326 (Table S7).

**Figure 2:**
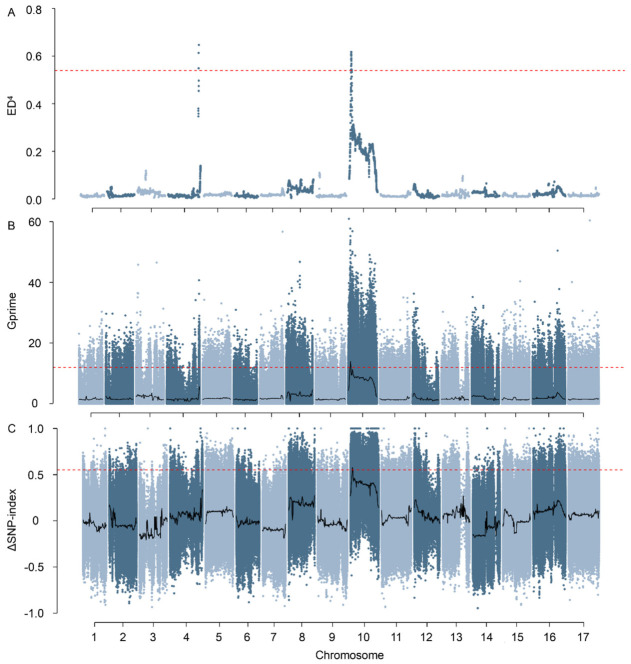
Identification of the major-effect locus *qBr10* on chromosome 10 using BSA-Seq. Manhattan plots displaying the results of three complementary statistical methods for bulk segregant analysis: (**A**) Euclidean Distance (ED), (**B**) G′ value, and (**C**) ΔSNP-index (Index-slid). The x-axis represents the physical position across the 17 sunflower chromosomes. The y-axis indicates the association statistic for each method. A significant, shared peak was consistently identified on chromosome 10. Horizontal dashed lines indicate the genome-wide threshold values used for candidate interval detection, namely 0.552 for Index-slid, 11.918 for G′, and 0.539 for ED^4^. Candidate regions were further required to contain at least 10 variants above the threshold.

By using ED, G′ value, and Index-slid methods, a significant signal was identified on chromosome 10 (Figure 2A–C), but the candidate intervals they reported varied in size (Table 2). The ED, G′, and Index-slid intervals spanned approximately 3.36 Mb, 5.38 Mb, and 3.40 Mb, respectively. Notably, the Index-slid interval was fully contained within the broader G′ interval and overlapped 94.1% of the ED interval, indicating that it captured the core signal supported by all three methods. In addition, Index-slid retained a similar number of effective variants as ED (149 vs. 148 effective SNPs; 19 vs. 19 effective InDels), while reducing the candidate gene set from 142 under G′ to 75 (Table 2). On this basis, the Index-slid interval was chosen as the main candidate area for further fine-mapping, and the significant quantitative trait locus (QTL) was named *qBr10*.

### 2.3. Fine-Mapping Narrows qBr10 to a 388.5 kb Interval

**Figure 3:**
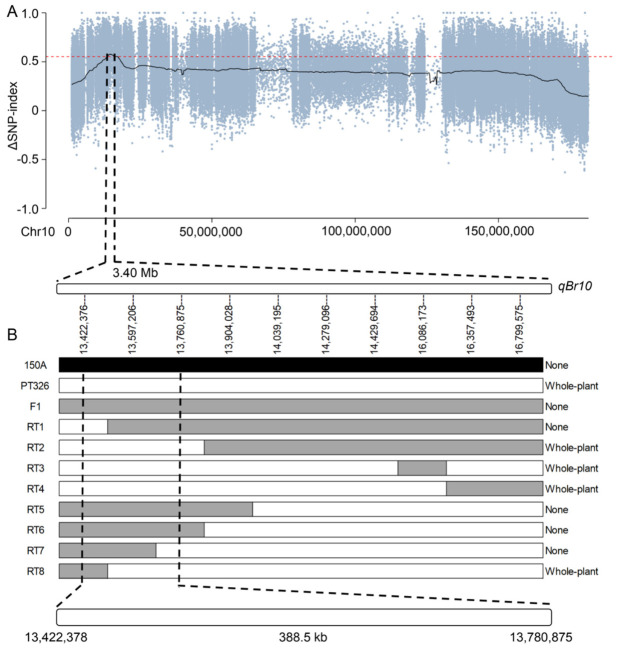
Fine-mapping of *qBr10* to a 388.5 kb interval on chromosome 10. (**A**) The initial candidate region for *qBr10* on chromosome 10, spanning approximately 3.40 Mb, as defined by the Index-slid method (Chr10:13,400,050–16,799,575 bp). Red dashed lines indicate the threshold values of 0.552 used for candidate interval detection for Index-slid. Black bars below represent the 10 Kompetitive Allele-Specific PCR (KASP) markers developed for recombinant screening. (**B**) Graphical genotypes of nine key recombinant F_2_ individuals (RT1–RT9) and their corresponding branching phenotypes (None or Whole-plant branching). Recombinant breakpoints are shown with parental 150A (non-branched) alleles in black, PT326 (branched) alleles in white, and heterozygous regions in gray. By aligning the genotypes with phenotypes, the *qBr10* locus was delimited to a 388.5 kb region between markers Chr10:13,422,378 and Chr10:13,780,875.

The significant QTL *qBr10* was located in a roughly 3.40 Mb region between Chr10:13,400,050 and Chr10:16,799,575. (Figure 3A). To enhance the resolution of *qBr10*, 10 KASP markers were created within this region (Table S1) and used to genotype the F_2_ mapping population. The uniformly non-branching phenotype of the F1 plants further supported the conclusion that non-branching is the dominant phenotype in this cross. In the F_2_ population of 660 individuals, nine recombinants with partial heterozygosity in the target region were identified as informative. RT2, RT3, RT4, and RT8 exhibited branching throughout the entire plant, while RT1, RT5, RT6, and RT7 did not show any branching. Through the comparison of recombinant breakpoints and their corresponding phenotypes, *qBr10* was narrowed down to a 388.5 kb physical region between markers Chr10:13,422,378 and Chr10:13,780,875 (Figure 3B).

### 2.4. Candidate Gene Analysis for qBr10

**Figure 4:**
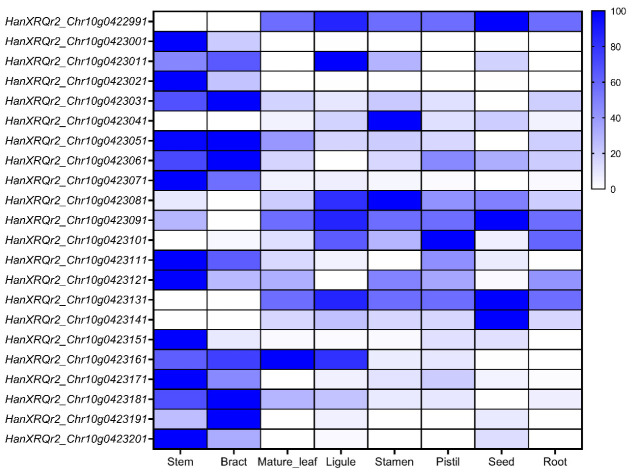
Expression profiles of candidate genes within the *qBr10* interval. Heatmap displaying the relative expression levels of the 21 genes located in the *qBr10* fine-mapped interval across various sunflower tissues, based on data from the SunExpress database. The expression scale ranges from low (white) to high (blue).

In the *qBr10* interval, a total of 21 annotated genes were identified (Table 3), which include predicted proteins with various functions, such as hypothetical proteins, enzymes, and regulatory proteins, such as fructokinase-1, glutathione S-transferase, pentatricopeptide repeat (PPR) proteins, histone H1 variants, and multiple candidates with potential regulatory functions. The SunExpress database was used to analyze expression profiles across tissues to prioritize candidates (Figure 4), highlighting stem expression because of the developmental beginnings of branch initiation and expansion. By integrating functional annotations and tissue-specific expression profiles, *HanXRQr2_Chr10g0423121* and *HanXRQr2_Chr10g0423031*, identified as transcription factor *WRKY21* and *MTB3*, were prioritized as the leading candidate gene for *qBr10*.

To provide additional sequence-based support for this prioritization, we further compared parental polymorphisms within and flanking these two genes (Table S8). *MTB3* harbored 33 polymorphisms between 150A and PT326, including one missense variant, one conservative in-frame insertion, two synonymous variants, and multiple intronic and upstream variants. *WRKY21* contained 45 polymorphisms, including one missense variant, two synonymous variants, four 5′-UTR variants, six 3′-UTR variants, and multiple intronic and flanking variants. These results provide additional comparative evidence for prioritizing *MTB3* and *WRKY21*, although they do not by themselves establish causality.

## 3. Discussion

The F_2_ segregation for None vs. Whole-plant branching aligned with a single major-effect locus where the non-branched trait is dominant. This inheritance differs from traditional sunflower studies, where branching is influenced by recessive alleles, such as the historic recessive branching locus identified by Putt in 1964 and later inheritance studies in cultivated germplasm [15]. The divergence in dominance relationships is not unexpected in sunflowers because branching has been repeatedly manipulated during breeding: unbranched A-lines and branching restorer lines have distinct selection histories, and different genetic sources of branching can segregate in different backgrounds [6,7,16]. Although PT326 displays a highly branched phenotype reminiscent of wild-type sunflower architecture, PT326 is a branched restorer line derived from cultivated germplasm rather than a wild accession. The major-effect segregation observed likely reflected the breeding history of this specific cultivated cross rather than a simple wild-versus-domesticated comparison. Overall, these findings back a model where sunflower branching is genetically diverse, with both dominant and recessive genes playing a role based on the source of the germplasm and how the trait is defined.

We also acknowledged that reducing the six branching categories to a binary classification for segregation analysis results in some loss of phenotypic information. The intermediate categories may contain biologically meaningful variation related to variable expressivity of the major locus, modifier loci, or environmental responsiveness. Therefore, the binary analysis used here is most appropriate for testing the presence of a major-effect locus and for maximizing phenotypic contrast in BSA-seq, but it may underestimate the contribution of additional loci affecting branching intensity or spatial distribution.

Three separate statistics (ED, G′, and Index-slid) all pointed to chromosome 10, suggesting that the signal is not due to a single metric. The G′ framework is especially useful as it explicitly accounts for sampling variance from both bulk construction and sequencing depth, then enhances robustness for real-world datasets by reducing noise through localized smoothing [13,17]. Moreover, each bulk contained 30 F_2_ individuals selected from the two most extreme and phenotypically unambiguous classes. This sample size falls within the 20–50-individual range commonly used in QTL-seq/BSA-seq and represents a practical compromise between maximizing phenotypic contrast and maintaining sequencing efficiency [18]. In the present study, the use of balanced extreme bulks and concordant signals across ED, G′, and Index-slid analyses supports that this design was sufficient for detecting the major-effect locus *qBr10*.

Environmental effects on branching should also be considered when interpreting *qBr10*. Although branching in sunflowers is known to be influenced by developmental and environmental conditions, the present study evaluated the trait in a single F_2_ population under one test environment. Therefore, the environmental stability of *qBr10* could not be determined from the current data. It remains possible that the phenotypic effect size, expressivity, or penetrance of *qBr10* may vary across locations, years, or cultivation conditions. Accordingly, *qBr10* should presently be regarded as a major-effect locus detected in this specific genetic background and test environment.

To ensure credibility, the BSA interval was not considered the final endpoint. KASP assays for marker-based fine-mapping reduced the *qBr10* region from several megabases to 388.5 kb. Nevertheless, the resolution of fine-mapping in the present study was constrained by the limited number of informative recombinants recovered in the target interval. Only nine recombinant F_2_ individuals were identified, which was sufficient to delimit *qBr10* to a 388.5 kb region but may not have provided enough breakpoint diversity to reduce the interval further. As a result, multiple annotated genes remained within the final interval, and the possibility of closely linked loci or additional local variation cannot yet be excluded. Therefore, further refinement of *qBr10* will require larger segregating populations, denser markers, and ultimately functional validation of prioritized candidate genes.

Sunflower branching has been examined through QTL mapping and association analyses, which often uncover polygenic regulation and distinct effects on apical and basal branching [4,6,7,12]. In this study, *qBr10* was identified as a major locus controlling whole-plant branching in this cross, providing a valuable starting point for gene cloning and functional validation. This was particularly notable for domestication-syndrome traits in sunflower, which typically lack large-effect QTL in conventional genetic crosses [4,12]. Previous studies have mapped the recessive apical branching gene b1 using marker-assisted strategies [19]. Importantly, the chromosome 10 region identified here is also broadly consistent with earlier reports that implicated the classical branching-related region on LG10/chromosome 10 in sunflower branching variation [6,7,12]. This suggests that *qBr10* may have broader relevance beyond the present cross and may represent a recurrent hotspot for branching-related variation in sunflower germplasm. However, because we did not perform direct marker collinearity or interval-level comparisons with previously reported loci, we cannot conclude that *qBr10* is identical to those earlier signals. Our results suggested that *qBr10* defined a precise physical interval at the gene scale for a dominant non-branching trait in the 150A × PT326 cross, while also highlighting its potential value for breeding efforts focused on capitulum number regulation and the improvement of ornamental multi-headed sunflower types.

The strong effect of *qBr10* should be interpreted in the context of the specific parental combination used here. Although PT326 displays a highly branched phenotype resembling the multi-headed architecture often associated with less-selected sunflower forms, its precise pedigree could not be confirmed from the available records used in this study. In addition, PT326 showed higher numbers of annotated genome-wide variants than 150A (Table S5). However, these values reflect relative sequence divergence from the reference genome rather than a direct measure of breeding history or within-line genetic heterogeneity. Accordingly, the identification of *qBr10* as a major-effect locus should be interpreted as a feature of the 150A × PT326 cross, rather than as evidence that branching in sunflower is generally controlled by a single locus.

In the narrowed *qBr10* region, there were several genes encoding enzymes such as fructokinase-1, detoxification-associated proteins like glutathione S-transferase, RNA-binding proteins involved in organellar RNA metabolism (PPR proteins), chromatin regulators including histone H1 variants, as well as diverse regulatory proteins such as transcription factors and ubiquitin-related factors. Although these gene families are involved in fundamental biological processes, they were not typically considered primary determinants of branching architecture. To avoid overinterpretation, we prioritized candidates using a comparative framework based on regulatory annotation, stem-related expression, and plausible relevance to plant architecture. Among the 21 annotated genes, only a limited subset encodes putative regulatory factors, including *MTB3*, *WRKY21*, *HanXRQr2_Chr10g0423061* (a putative CCHC(Zn) family regulator), and *HanXRQr2_Chr10g0423131* (an F-box/kelch-repeat protein). In contrast, many of the remaining genes encode proteins associated with general metabolism, chromatin structure, organellar function, transport, or poorly characterized activities. In addition to expression-based prioritization, both genes also showed coding and non-coding polymorphisms between the parental lines, which strengthens their candidacy at the sequence level. On this basis, *WRKY21* and *MTB3* were treated as higher-priority candidates rather than exclusive causal genes, while other genes in the interval remain possible contributors until excluded by additional evidence.

However, the current candidate gene prioritization should be interpreted with caution. Although annotation and tissue-expression profiles provide useful evidence for narrowing the candidate list, they are not sufficient to establish causality. In the present study, *WRKY21* and *MTB3* were prioritized only as plausible candidate genes within the fine-mapped *qBr10* interval, and no direct functional validation was performed. Therefore, the present data support candidate prioritization rather than definitive gene identification. Future work should combine higher-resolution expression analysis and functional assays to determine whether either of these genes is the causal determinant of the *qBr10* phenotype.

As plant-specific transcription factors, the WRKY family coordinates developmental programs with environmental and hormonal signals. These regulators frequently operate as transcriptional switches, thereby allowing growth patterns to be dynamically adjusted according to shifts in internal physiological conditions [20,21]. For architectural traits like branching, such regulators represent credible causal candidates, because branching decisions arise from transcriptional reprogramming within axillary buds and the surrounding stem tissues. Our results suggested that *WRKY21* could alter the balance between axillary bud dormancy and activation via modifying expression levels, DNA-binding specificity, or responsiveness to upstream signals, thereby converting plants from a non-branching phenotype to a whole-plant branching phenotype.

*MTB3* is annotated as a transcription factor and thus matches the expectation that major architectural loci often encode regulatory proteins rather than metabolic enzymes. Notably, transcription-factor modules that mediate stress–growth tradeoffs (including jasmonate-centered signaling) can influence axillary bud activity indirectly through resource allocation and hormonal cross-talk. While jasmonate signaling is primarily studied for defense, it also modulates growth and reproductive development, and bHLH-centered networks are recurrent motifs in these pathways [22,23,24]. We suggested *MTB3* could provide a mechanistically coherent second candidate, especially when combined with stem-biased expression evidence.

Branching is a developmentally complex trait regulated by multiple interacting pathways, including auxin-, cytokinin-, and strigolactone-mediated control of axillary bud outgrowth, as well as transcriptional regulators such as *TB1*/*BRC1* [25,26]. In this study, *qBr10* was identified as a major-effect locus affecting branching architecture, but the current data do not allow us to place it definitively within a known signaling pathway. Nevertheless, the prioritized candidate genes *WRKY21* and *MTB3* may be biologically relevant to developmental and regulatory processes associated with branch formation. We therefore interpret *qBr10* as a locus that may participate in known branching regulatory networks, while recognizing that its precise mechanistic role remains unresolved and requires further functional study.

## 4. Materials and Methods

### 4.1. Plant Materials and Growth Conditions

Two inbred lines with contrasting architecture, 150A (monocephalic, non-branched) and PT326 (polycephalic, highly branched), were used. F_1_ plants were created by crossing 150A with PT326 and then self-pollinated to develop an F_2_ population of 660 individuals for segregation analysis, BSA mapping, and fine-mapping. In the 2025 cropping season, field trials were conducted at Shenyang (123.5 E, 41.8 N) in Liaoning Province, China. The parental lines and F_1_ were planted in a randomized complete block design with three biological replicates. Each plot was planted in a two-row plot with a row length of 6 m. For the segregating F_2_ population, 660 individuals were also planted in the same field for phenotyping and genotyping-based mapping. Seedlings were spaced 60 cm between plants and 50 cm between rows. Protection lines were consistently set up across the field, following local sunflower production standards for field management. Growth stages and phenotyping were scheduled according to the conventional sunflower developmental scale [27].

### 4.2. Phenotyping and Genetic Analysis

Branching phenotype was evaluated at the flowering stage, when lateral branches were fully visible and could be reliably distinguished from small axillary buds. For scoring consistency, the main stem was divided into three equal sections according to relative plant height: basal (0–33% of stem height from the stem base), middle (34–66%), and apical (67–100%, excluding the terminal capitulum). A lateral branch was recorded only when the axillary shoot was visibly elongated to more than 2 cm. Based on the distribution of elongated branches along the main stem, each F_2_ individual was classified as None, Basal, Mid-basal, Mid-apical, Apical, or Whole-plant branching. For the segregation analysis and BSA-seq, we used only the two extreme and phenotypically unambiguous classes, namely None and Whole-plant branching, and excluded the intermediate categories from the binary genetic test. A chi-square goodness-of-fit test was used to compare the observed counts with the expected 3:1 Mendelian segregation ratio. The chi-square statistic, degrees of freedom (*df* = 1), and the exact *p*-value were reported. In addition, the observed proportion of Whole-plant branching individuals was summarized with a 95% confidence interval, and segregation distortion was evaluated based on deviation from the expected ratio.

### 4.3. DNA Extraction, Bulk Construction, and Resequencing

Genomic DNA was isolated from the young leaves of both parents and selected F_2_ individuals using a Cetyltrimethylammonium Bromide (CTAB) protocol [28]. For bulk construction, 30 F_2_ individuals from each of the two most extreme and phenotypically unambiguous classes (None and Whole-plant branching) were selected to form the two DNA bulks. This sample size was chosen to balance phenotypic contrast, pool balance, and sequencing efficiency. According to QTL-seq recommendations, DNA concentration and quality were determined through gel electrophoresis and fluorometric analysis [18]; two bulks were created by mixing equal DNA amounts from 30 None-type and 30 Whole-plant-type F_2_ individuals, respectively. For parents and bulks, paired-end Illumina libraries were constructed, and fastp was used to process raw reads for quality control and trimming of adapters [29].

### 4.4. Read Alignment, Variant Calling, and Annotation

Raw reads were further filtered to remove adapter contamination and low-quality bases, and subsequently aligned to the sunflower reference genome (HanXRQr2) using BWA-MEM [30]. Alignments were processed with SAMtools [31] to minimize PCR-induced bias; duplicate reads were marked. Variants, including SNPs and InDels, were identified using the Genome Analysis Toolkit (GATK) best-practices framework [32,33]. Only biallelic SNPs and InDels were kept. Variants were required to meet the following thresholds: variant quality score (QUAL) ≥30, mapping quality (MQ) ≥30, and sufficient read support in all samples. Specifically, a minimum read depth of 10 was required in each parent, and a minimum read depth of 15 was required in each bulk. Sequencing quality was evaluated using clean data yield, Q30, mapping rate, average sequencing depth, and genome coverage. Coverage uniformity was assessed based on the breadth and consistency of reference-genome coverage across the parental and bulked samples. The filtering process eliminated low-confidence sites and preserved biallelic variants with enough read depth in both bulks. For BSA statistics, only markers that were parentally informative and fixed for different homozygous alleles between 150A and PT326 were retained, following the QTL-seq approach to enhance signal clarity [18]. Variant effect prediction was performed with SnpEff [34]. Variant data were managed in VCF format and filtered with VCFtools where needed [35].

### 4.5. BSA Mapping and Candidate Interval Definition

BSA follows the classical framework of Michelmore et al. [36]. In order to maximize power, we preserved biallelic, high-quality variants that were informative for parents and fixed for different homozygous alleles between the parents. Using complementary statistics, genome-wide scans were executed, utilizing SNP-index/Δ(SNP-index) in sliding windows as per the QTL-seq methodology [18]. For the Index-slid analysis, the Δ(SNP-index) values were smoothed using a sliding-window approach to reduce stochastic noise, and the 0.999 quantile of the genome-wide Index-slid distribution was used as the significance threshold. The corresponding cutoff value was 0.552. The G′ statistic is a refined form of the G statistic, designed for sequencing-based BSA, which considers read-depth sampling variance and linkage structure [17]. Smoothed G′ values were calculated using a 1 Mb sliding window, and the 0.999 quantile of the genome-wide G′ distribution was used as the screening threshold. The corresponding cutoff value was 11.918. Euclidean distance (ED) between allele-frequency vectors of bulks serves as an extra, model-independent signal detector. The ED values were first raised to the fourth power to enhance signal discrimination, and the transformed values were then smoothed using a sliding-window approach with a 4 Mb window size and 200 kb step size. The 99.9th percentile of the genome-wide smoothed ED^4^ distribution was used as the significance threshold, corresponding to a cutoff value of 0.539. For each statistic, genome-wide profiles resembling Manhattan plots were produced. Only regions containing at least 10 variant sites above the threshold were retained as candidate intervals.

### 4.6. KASP Marker Development and Fine-Mapping

In the candidate region of major-effect QTL, KASP assays were carried out by using established principles of competitive allele-specific PCR [37]. Priority was given to SNPs with evident parental differences and stable surrounding sequences. Ten KASP markers were selected (Table S1) and confirmed using the DNA of parental and F_1_ plants. Genotyping was performed on all 660 F_2_ individuals, and recombinant individuals were identified by locating crossover breakpoints between the flanking markers. Recombinant genotypes in relation to branching characteristics were evaluated to narrow the interval of major-effect QTL.

### 4.7. Candidate Gene Analysis

After fine-mapping, the genes located in the interval were obtained from the reference annotation. The compilation of functional annotation involved predicted protein domains, homology-based descriptions, and classifications of gene families. To identify genes that could regulate branching, the tissue-specific expression data from different tissues in sunflower were obtained from the SunExpress expression database [7,38].

## 5. Conclusions

We identified *qBr10* as a major-effect QTL governing whole-plant shoot branching in sunflower by combining BSA-seq signals from multiple statistical methods and verifying the target locus via KASP genotyping in recombinant individuals, we successfully narrowed *qBr10* from a large multi-megabase candidate region down to a 388.5 kb interval on chromosome 10. This provides a clearly defined, marker-based target for further functional characterization and molecular breeding applications. Annotation of this interval revealed 21 genes of diverse functions; however, combining positional evidence with regulatory plausibility and stem-relevant expression patterns supports *HanXRQr2_Chr10g0423121* (*WRKY21*) and *HanXRQr2_Chr10g0423031* (*MTB3*) as the most likely causal candidates. Our results provided a focused foundation for functional validation and allele mining of branching regulators to advance sunflower improvement.

## Figures and Tables

**Table 1:** Chi-square (χ^2^) test for goodness-of-fit to a 3:1 segregation ratio in the F_2_ population.

Phenomenon	Expected Number of Plants	Observed Number of Plants	*df*	χ^2^ Test	χ^2^ (*p* = 0.05)
None	469	451	1	2.916	3.84
Whole-plant branching	156	175			

**Table 2:** Summary of candidate intervals identified by different BSA methods on chromosome 10.

Method	Candidate Interval	Interval Length (Mb)	Total SNPs	Effective SNPs	Total InDels	Effective InDels	Annotated Genes
Euclidean Distance (ED)	chr10:13,202,553:16,561,272	3.36	3616	148	566	19	74
G′	chr10:11,892,034:17,271,104	5.38	7401	217	1124	32	142
Index-slid	chr10:13,400,050:16,799,575	3.40	3702	149	583	19	75

**Table 3:** Curated putative annotations of the 21 genes within the fine-mapped *qBr10* interval (Chr10:13,422,378–13,780,875).

Gene ID	Curated Putative Annotation
*HanXRQr2_Chr10g0423001*	Uncharacterized protein (best NR homolog:HanPI659440_Chr10g0364921)
*HanXRQr2_Chr10g0423011*	putative nucleotidyltransferase, Ribonuclease H
*HanXRQr2_Chr10g0423021*	Uncharacterized protein (homologous to HanXRQr2_Chr10g0423001)
*HanXRQr2_Chr10g0423031*	transcription factor *MTB3*
*HanXRQr2_Chr10g0423041*	Uncharacterized protein
*HanXRQr2_Chr10g0423051*	fructokinase-1
*HanXRQr2_Chr10g0423061*	putative transcription factor interactor and regulator CCHC(Zn) family
*HanXRQr2_Chr10g0423071*	glutathione S-transferase T3
*HanXRQr2_Chr10g0423081*	pentatricopeptide repeat-containing protein At1g62930, chloroplastic
*HanXRQr2_Chr10g0423091*	putative Zeta toxin domain, P-loop containing nucleoside triphosphate hydrolase
*HanXRQr2_Chr10g0423101*	histone H1
*HanXRQr2_Chr10g0423111*	histone H1
*HanXRQr2_Chr10g0423121*	probable WRKY transcription factor 21
*HanXRQr2_Chr10g0423131*	F-box/kelch-repeat protein At1g57790
*HanXRQr2_Chr10g0423141*	putative plant self-incompatibility S1
*HanXRQr2_Chr10g0423151*	magnesium transporter MRS2-I
*HanXRQr2_Chr10g0423161*	photosystem II reaction center W protein, chloroplastic
*HanXRQr2_Chr10g0423171*	probable galacturonosyltransferase 6
*HanXRQr2_Chr10g0423181*	putative glutathione-disulfide reductase
*HanXRQr2_Chr10g0423191*	No informative annotation available
*HanXRQr2_Chr10g0423201*	Uncharacterized protein (best homolog: HanXRQr2_Chr01g0008831)

## Data Availability

The original contributions presented in the study are included in the article/Supplementary Material; further inquiries can be directed to the corresponding author.

## Supplementary Materials

The following supporting information can be downloaded at: https://www.mdpi.com/article/10.3390/ijms27093715/s1.
