# Supplementary material for: Identification and Fine-Mapping of qBr10, a Major-Effect Locus for Shoot Branching in Sunflower (Helianthus annuus)
Source: Int J Mol Sci. 2026 Apr 22;27(9):3715. doi: 10.3390/ijms27093715 (PMC13164398; doi:10.3390/ijms27093715)
Supplement: Supplementary file 1 [file ijms-27-03715-s001.zip › ijms-4218825-supplementary.pdf]

Table S1 Kompetitive Allele-Specific PCR (KASP) markers developed for fine-mapping of *qBr10* on chromosome 10.

Chr	Position	Primer_Allele X	Primer_Allele Y	Primer_Common
chr10	13422376	GAAGGTGACCAAGTTCATGCTCCGCTTAGTCTTTACTTAGGCTTCT	GAAGGTCGGAGTCAACGGATTCCGCTTAGTCTTTACTTAGGCTTCG	AACATGCCGAAATTGAAGCCACTAC
chr10	13597206	GAAGGTGACCAAGTTCATGCTCCAAAGGTTTAAGAACATGCCCGA	GAAGGTCGGAGTCAACGGATTCCAAAGGTTTAAGAACATGCCCGG	TCTACTTGGGAGGGTTTTGACCAC
chr10	13760875	GAAGGTGACCAAGTTCATGCTGTCACCGTATTCTTCTATCGCCTGC	GAAGGTCGGAGTCAACGGATTGTCACCGTATTCTTCTATCGCCTGA	AGTCGGTGTTAGTGAGCAACAGGTG
chr10	13904028	GAAGGTGACCAAGTTCATGCTAGCTTGACGACTTCCATCTCATAAC	GAAGGTCGGAGTCAACGGATTAGCTTGACGACTTCCATCTCATAAG	AAGGACTTTGGAAATCACCTTGCTG
chr10	14039195	GAAGGTGACCAAGTTCATGCTGACTAAAACAAGCAGCCAAATCTCT	GAAGGTCGGAGTCAACGGATTGACTAAAACAAGCAGCCAAATCTCA	CCGATACGATAATTTGGGCTTTCAA
chr10	14279096	GAAGGTGACCAAGTTCATGCTGTCCCATTCGACAATGCTTTTCC	GAAGGTCGGAGTCAACGGATTGTCCCATTCGACAATGCTTTTCT	GAAGAAGGAGAATAAAACGTGGGTA
chr10	14429694	GAAGGTGACCAAGTTCATGCTTCGAACCTCTGCTATCAAGCGAAA	GAAGGTCGGAGTCAACGGATTTCGAACCTCTGCTATCAAGCGAAG	TTCCGACATCATCTTTGAAAACGAG
chr10	16086173	GAAGGTGACCAAGTTCATGCTATGGAAATCAATAGTGCCTTCTTCA	GAAGGTCGGAGTCAACGGATTATGGAAATCAATAGTGCCTTCTTCG	AATGAAGAGGTCCAAGAAATGATGC
chr10	16357493	GAAGGTGACCAAGTTCATGCTGTCAATTCACGGTTGCATCATGTT	GAAGGTCGGAGTCAACGGATTGTCAATTCACGGTTGCATCATGTC	CCACTTGACGCTAGACGGAATTGTA
chr10	16799575	GAAGGTGACCAAGTTCATGCTTTCACGGTACAAATTTAAAGGTCCA	GAAGGTCGGAGTCAACGGATTTTCACGGTACAAATTTAAAGGTCCG	TTCCTTGAACTATAACCGTGGATGC

Abbreviations: KASP, Kompetitive Allele-Specific PCR; Chr, chromosome.

Table S2 Summary of sequencing data quality for parental lines and extreme bulks.

Sample ID	Raw Reads	Raw Bases（bp）	Raw GC（%）	Raw Q30（%）	Clean Reads	Clean Bases（bp）	Clean GC（%）	Clean Q30（%）	Mapped Ratio(%)	Proper Ratio(%)	Insert Size	Real Depth	Coverage(%) (>=1x)	Coverage(%) (>=4x)
150A	250,850,150	75,756,745,300	38.99	96.42	249,420,611	75,217,208,462	38.98	96.8	99.81	95.9	302	27.55	90.55	87.47
PT326	249,694,485	75,407,734,470	38.67	96.38	247,946,239	74,763,693,206	38.61	96.83	99.69	93.1	293	28.34	87.37	82.22
None type	359,787,034	108,655,684,268	38.82	96.48	357,529,378	107,818,286,768	38.8	96.93	99.74	94.35	308	37.76	94.63	92.66
Whole plant type	339,999,203	102,679,759,306	38.78	95.86	337,631,052	101,808,919,604	38.74	96.3	99.73	94.48	297	35.66	94.61	92.64

Abbreviations: bp, base pairs; GC, guanine-cytosine content; Q30, proportion of bases with a Phred quality score ≥30.

Table S3 Genome-wide single nucleotide polymorphism (SNP) statistics for each sample.

Sample ID	SNP Number	Transition	Transversion	Ts/Tv	Heterozygosity Number	Homozygosity Number
150A	5,782,892	3,754,982	2,027,910	1.85	1,860,967	12,406,682
PT326	10,326,094	6,685,038	3,641,056	1.84	3,331,991	10,742,863
None type	12,016,108	7,778,522	4,237,586	1.84	9,624,515	5,334,591
Whole-plant branching type	12,178,568	7,880,280	4,298,288	1.83	9,792,067	5,169,688

Abbreviations:SNP, single nucleotide polymorphism; Ts/Tv, transition/transversion ratio.

Table S4 Genome-wide insertion-deletion (InDel) statistics for each sample.

Sample ID	Insert Number	Delete Number	Heterozygosity Number	Homozygosity Number
150A	444,758	480,590	228,835	696,513
PT326	764,887	833,617	390,537	1,207,967
None type	914,647	1,003,456	1,503,779	414,324
Whole-plant branching type	927,490	1,018,663	1,533,674	412,479

Abbreviations: InDel, insertion-deletion polymorphism.

Table S5 Functional annotation of genome-wide SNPs.

Sample ID	Start Lost	Stop Lost	Stop Gained	Missense Variant	Synonymous Variant	Intragenic Region
150A	472	560	2,272	104,965	124,570	4,953,832
PT326	724	924	4,080	183,542	218,397	8,864,004
None type	899	1,097	4,861	220,435	266,073	10,241,993
Whole-plant branching type	907	1,103	4,919	222,411	267,835	10,387,718

Abbreviations: SNP, single nucleotide polymorphism.

Table S6 Functional annotation of genome-wide InDels.

Sample ID	Frameshift Variant	Intergenic Region	Intragenic Variant	Start Lost	Stop Gained	Stop Lost
150A	8,674	737,263	0	185	313	171
PT326	15,606	1,267,608	0	398	576	310
None type	17,953	1,511,391	0	453	618	385
Whole-plant branching type	18,123	1,535,231	0	459	621	383

Abbreviations: InDel, insertion-deletion polymorphism.

Table S7 Summary of filtered, parentally informative markers used for BSA association analysis.

Chrom	SNP Number	Effective SNP	InDel Number	Effective InDel
chr1	173,605	5,079	29,064	597
chr2	241,810	5,317	33,994	628
chr3	115,705	3,219	19,384	363
chr4	141,266	3,835	22,780	472
chr5	319,791	6,821	44,613	780
chr6	145,061	3,708	23,725	444
chr7	168,613	3,886	25,583	433
chr8	176,655	4,393	28,596	582
chr9	225,612	5,817	36,487	731
chr10	237,592	5,476	36,101	657
chr11	359,639	7,070	49,143	738
chr12	104,900	3,155	19,536	416
chr13	168,028	3,411	24,728	389
chr14	225,771	6,067	32,847	691
chr15	401,747	8,765	58,673	1,092
chr16	262,410	5,666	39,459	706
chr17	323,931	6,258	45,640	759

Abbreviations: BSA, bulked segregant analysis; SNP, single nucleotide polymorphism; InDel, insertion-deletion polymorphism.

Table S8 SNP and small InDel polymorphisms detected between the parental lines 150A and PT326 within and flanking the prioritized candidate genes *MTB3* and *WRKY21* in the *qBr10* interval.

Chr	Position	Variant type	Genotype in 150A	Genotype in PT326	Functional class	PutativeImpact	Gene ID	HGVS.c	HVGS.p
chr10	13445582	INDEL	A/A	AACC/AACC	conservative_inframe_insertion	MODERATE	HanXRQr2_Chr10g0423031	c.730_732dupGGT	p.Gly244dup
chr10	13445673	SNP	A/A	G/G	synonymous_variant	LOW	HanXRQr2_Chr10g0423031	c.642T>C	p.Phe214Phe
chr10	13445736	SNP	A/A	G/G	synonymous_variant	LOW	HanXRQr2_Chr10g0423031	c.579T>C	p.Val193Val
chr10	13445887	SNP	T/T	A/A	missense_variant	MODERATE	HanXRQr2_Chr10g0423031	c.428A>T	p.Tyr143Phe
chr10	13446399	INDEL	ATATTAAT/ATATTAAT	A/A	intron_variant	MODIFIER	HanXRQr2_Chr10g0423031	c.-2-84_-2-83insATTAATA	--
chr10	13446525	INDEL	AC/AC	A/A	intron_variant	MODIFIER	HanXRQr2_Chr10g0423031	c.-2-210_-2-209insG	--
chr10	13446544	INDEL	GA/GA	G/G	intron_variant	MODIFIER	HanXRQr2_Chr10g0423031	c.-2-229dupT	--
chr10	13446656	SNP	C/C	T/T	intron_variant	MODIFIER	HanXRQr2_Chr10g0423031	c.-3+148A>G	--
chr10	13446668	SNP	C/C	T/T	intron_variant	MODIFIER	HanXRQr2_Chr10g0423031	c.-3+136A>G	--
chr10	13447124	SNP	T/T	A/A	upstream_gene_variant	MODIFIER	HanXRQr2_Chr10g0423031	c.-323A>T	--
chr10	13447599	INDEL	C/C	CATTT/CATTT	upstream_gene_variant	MODIFIER	HanXRQr2_Chr10g04	c.-799_-798insAAAT	--

							23031		
chr10	13447620	SNP	T/T	A/A	upstream_gene_variant	MODIFIER	HanXRQr2_Chr10g0423031	c.-819A>T	--
chr10	13447653	INDEL	TGA/TGA	T/T	upstream_gene_variant	MODIFIER	HanXRQr2_Chr10g0423031	c.-854_-853delTC	--
chr10	13447671	INDEL	GAA/GAA	G/G	upstream_gene_variant	MODIFIER	HanXRQr2_Chr10g0423031	c.-872_-871delTT	--
chr10	13447687	SNP	A/A	G/G	upstream_gene_variant	MODIFIER	HanXRQr2_Chr10g0423031	c.-886T>C	--
chr10	13447707	SNP	T/T	C/C	upstream_gene_variant	MODIFIER	HanXRQr2_Chr10g0423031	c.-906A>G	--
chr10	13447855	SNP	C/C	T/T	upstream_gene_variant	MODIFIER	HanXRQr2_Chr10g0423031	c.-1054G>A	--
chr10	13447883	SNP	C/C	T/T	upstream_gene_variant	MODIFIER	HanXRQr2_Chr10g0423031	c.-1082G>A	--
chr10	13447908	SNP	T/T	C/C	upstream_gene_variant	MODIFIER	HanXRQr2_Chr10g0423031	c.-1107A>G	--
chr10	13447946	INDEL	CA/CA	C/C	upstream_gene_variant	MODIFIER	HanXRQr2_Chr10g0423031	c.-1146delT	--
chr10	13447964	SNP	A/A	G/G	upstream_gene_variant	MODIFIER	HanXRQr2_Chr10g0423031	c.-1163T>C	--
chr10	13447971	SNP	C/C	A/A	upstream_gene_variant	MODIFIER	HanXRQr2_Chr10g0423031	c.-1170G>T	--
chr10	13448029	SNP	C/C	A/A	upstream_gene_variant	MODIFIER	HanXRQr2_Chr10g0423031	c.-1228G>T	--
chr10	13448144	SNP	A/A	G/G	upstream_gene_variant	MODIFIER	HanXRQr2_Chr10g04	c.-1343T>C	--

							23031		
chr10	13448212	INDEL	G/G	GA/GA	upstream_gene_variant	MODIFIER	HanXRQr2_Chr10g0423031	c.-1412_-1411insT	--
chr10	13448268	SNP	A/A	G/G	upstream_gene_variant	MODIFIER	HanXRQr2_Chr10g0423031	c.-1467T>C	--
chr10	13448278	INDEL	G/G	GTATCGTGT/GTATCGTGT	upstream_gene_variant	MODIFIER	HanXRQr2_Chr10g0423031	c.-1478_-1477insACACGATA	--
chr10	13448290	SNP	T/T	C/C	upstream_gene_variant	MODIFIER	HanXRQr2_Chr10g0423031	c.-1489G>A	--
chr10	13448448	INDEL	G/G	GA/GA	upstream_gene_variant	MODIFIER	HanXRQr2_Chr10g0423031	c.-1648_-1647insT	--
chr10	13448490	SNP	T/T	A/A	upstream_gene_variant	MODIFIER	HanXRQr2_Chr10g0423031	c.-1689A>T	--
chr10	13448556	SNP	C/C	T/T	upstream_gene_variant	MODIFIER	HanXRQr2_Chr10g0423031	c.-1755G>A	--
chr10	13448582	INDEL	T/T	TTATGCA/TTATGCA	upstream_gene_variant	MODIFIER	HanXRQr2_Chr10g0423031	c.-1782_-1781insTGCATA	--
chr10	13448924	SNP	C/C	T/T	upstream_gene_variant	MODIFIER	HanXRQr2_Chr10g0423031	c.-2123G>A	--
chr10	13603518	INDEL	CAA/CAA	C/C	downstream_gene_variant	MODIFIER	HanXRQr2_Chr10g0423121	c.*1921_*1922delTT	--
chr10	13603590	SNP	G/G	A/A	downstream_gene_variant	MODIFIER	HanXRQr2_Chr10g0423121	c.*1851C>T	--
chr10	13603808	SNP	G/G	T/T	downstream_gene_variant	MODIFIER	HanXRQr2_Chr10g0423121	c.*1633C>A	--
chr10	13603811	SNP	T/T	C/C	downstream_gene_varian	MODIFIER	HanXRQr2_Chr10g04	c.*1630A>G	--

					t		23121		
chr10	13603902	SNP	A/A	G/G	downstream_gene_variant	MODIFIER	HanXRQr2_Chr10g0423121	c.*1539T>C	--
chr10	13603917	SNP	G/G	A/A	downstream_gene_variant	MODIFIER	HanXRQr2_Chr10g0423121	c.*1524C>T	--
chr10	13604023	SNP	A/A	C/C	downstream_gene_variant	MODIFIER	HanXRQr2_Chr10g0423121	c.*1418T>G	--
chr10	13604044	SNP	T/T	A/A	downstream_gene_variant	MODIFIER	HanXRQr2_Chr10g0423121	c.*1397A>T	--
chr10	13604772	SNP	T/T	G/G	downstream_gene_variant	MODIFIER	HanXRQr2_Chr10g0423121	c.*669A>C	--
chr10	13604793	SNP	T/T	A/A	downstream_gene_variant	MODIFIER	HanXRQr2_Chr10g0423121	c.*648A>T	--
chr10	13604815	SNP	C/C	G/G	downstream_gene_variant	MODIFIER	HanXRQr2_Chr10g0423121	c.*626G>C	--
chr10	13604832	SNP	G/G	C/C	downstream_gene_variant	MODIFIER	HanXRQr2_Chr10g0423121	c.*609C>G	--
chr10	13604833	SNP	T/T	C/C	downstream_gene_variant	MODIFIER	HanXRQr2_Chr10g0423121	c.*608A>G	--
chr10	13605228	INDEL	A/A	AT/AT	3_prime_UTR_variant	MODIFIER	HanXRQr2_Chr10g0423121	c.*212dupA	--
chr10	13605235	INDEL	A/A	AAAAC/AAAAC	3_prime_UTR_variant	MODIFIER	HanXRQr2_Chr10g0423121	c.*205_*206insGTTT	--
chr10	13605284	SNP	T/T	C/C	3_prime_UTR_variant	MODIFIER	HanXRQr2_Chr10g0423121	c.*157A>G	--
chr10	13605333	SNP	T/T	C/C	3_prime_UTR_variant	MODIFIER	HanXRQr2_Chr10g04	c.*108A>G	--

							23121		
chr10	13605366	SNP	C/C	T/T	3_prime_UTR_variant	MODIFIER	HanXRQr2_Chr10g0423121	c.*75G>A	--
chr10	13605431	INDEL	GT/GT	G/G	3_prime_UTR_variant	MODIFIER	HanXRQr2_Chr10g0423121	c.*9delA	--
chr10	13606222	SNP	T/T	C/C	intron_variant	MODIFIER	HanXRQr2_Chr10g0423121	c.899+81A>G	--
chr10	13606443	SNP	A/A	C/C	intron_variant	MODIFIER	HanXRQr2_Chr10g0423121	c.774-15T>G	--
chr10	13606455	SNP	A/A	C/C	intron_variant	MODIFIER	HanXRQr2_Chr10g0423121	c.774-27T>G	--
chr10	13606468	INDEL	TTGTAATAC/TTGTAATAC	T/T	intron_variant	MODIFIER	HanXRQr2_Chr10g0423121	c.774-48_774-41delGTATTACA	--
chr10	13606563	SNP	G/G	A/A	intron_variant	MODIFIER	HanXRQr2_Chr10g0423121	c.774-135C>T	--
chr10	13606566	SNP	T/T	A/A	intron_variant	MODIFIER	HanXRQr2_Chr10g0423121	c.774-138A>T	--
chr10	13606620	SNP	T/T	A/A	intron_variant	MODIFIER	HanXRQr2_Chr10g0423121	c.774-192A>T	--
chr10	13606633	SNP	C/C	T/T	intron_variant	MODIFIER	HanXRQr2_Chr10g0423121	c.774-205G>A	--
chr10	13606655	INDEL	C/C	CA/CA	intron_variant	MODIFIER	HanXRQr2_Chr10g0423121	c.774-228dupT	--
chr10	13606790	SNP	G/G	A/A	intron_variant	MODIFIER	HanXRQr2_Chr10g0423121	c.773+215C>T	--
chr10	13606793	SNP	C/C	A/A	intron_variant	MODIFIER	HanXRQr2_Chr10g04	c.773+212G>T	--

							23121		
chr10	13606829	SNP	T/T	C/C	intron_variant	MODIFIER	HanXRQr2_Chr10g0423121	c.773+176A>G	--
chr10	13606842	SNP	G/G	T/T	intron_variant	MODIFIER	HanXRQr2_Chr10g0423121	c.773+163C>A	--
chr10	13606862	SNP	C/C	T/T	intron_variant	MODIFIER	HanXRQr2_Chr10g0423121	c.773+143G>A	--
chr10	13606892	SNP	A/A	G/G	intron_variant	MODIFIER	HanXRQr2_Chr10g0423121	c.773+113T>C	--
chr10	13606905	SNP	T/T	C/C	intron_variant	MODIFIER	HanXRQr2_Chr10g0423121	c.773+100A>G	--
chr10	13606932	SNP	T/T	C/C	intron_variant	MODIFIER	HanXRQr2_Chr10g0423121	c.773+73A>G	--
chr10	13606985	SNP	C/C	T/T	intron_variant	MODIFIER	HanXRQr2_Chr10g0423121	c.773+20G>A	--
chr10	13607148	SNP	C/C	A/A	synonymous_variant	LOW	HanXRQr2_Chr10g0423121	c.630G>T	p.Thr210Thr
chr10	13607175	SNP	C/C	A/A	synonymous_variant	LOW	HanXRQr2_Chr10g0423121	c.603G>T	p.Ser201Ser
chr10	13607392	SNP	A/A	T/T	missense_variant	MODERATE	HanXRQr2_Chr10g0423121	c.386T>A	p.Ile129Asn
chr10	13608211	INDEL	GT/GT	G/G	5_prime_UTR_variant	MODIFIER	HanXRQr2_Chr10g0423121	c.-147delA	--
chr10	13608219	INDEL	TA/TA	T/T	5_prime_UTR_variant	MODIFIER	HanXRQr2_Chr10g0423121	c.-155delT	--
chr10	13608241	INDEL	TG/TG	T/T	5_prime_UTR_variant	MODIFIER	HanXRQr2_Chr10g04	c.-177delC	--

							23121		
chr10	13608264	INDEL	AC/AC	A/A	5_prime_UTR_variant	MODIFIER	HanXRQr2_Chr10g04 23121	c.-200delG	--
chr10	13608714	SNP	A/A	C/C	upstream_gene_variant	MODIFIER	HanXRQr2_Chr10g04 23121	c.-649T>G	--

Abbreviations: SNP, single nucleotide polymorphism; InDel, insertion-deletion polymorphism; HGVS.c, coding DNA sequence nomenclature according to Human Genome Variation Society guidelines; HGVS.p, protein sequence nomenclature according to Human Genome Variation Society guidelines; Chr, chromosome.
